# Factors associated with physical activity in individuals with metastatic cancer: a UK cross-sectional survey

**DOI:** 10.1007/s11764-024-01700-5

**Published:** 2024-10-28

**Authors:** Zainab Faatimah Haider, Samuel George Smith, Rebecca E. A. Walwyn, Phillippa Lally, Abigail Fisher, Rebecca J. Beeken

**Affiliations:** 1 Leeds Institute of Health Sciences, University of Leeds, Leeds, UK; 2 Leeds Institute of Clinical Trials Research, University of Leeds, Leeds, UK; 3 University of Surrey, Guildford, UK; 4 Institute of Epidemiology and Health Care, University College London, London, UK

**Keywords:** Physical activity, Metastatic cancer, Observational study, Physical activity support, Ethnic minority

## Abstract

**Purpose:**

Physical activity is safe and feasible for individuals with metastatic cancer and may support symptom management. We investigated the extent to which individuals with metastatic cancer are meeting the World Health Organisation (WHO) moderate-vigorous physical activity (MVPA) guideline, factors associated with meeting the guideline, and perceptions about physical activity and receiving physical activity advice.

**Methods:**

Data were from UK adults with metastatic breast, prostate, or colorectal cancer who completed the Healthy Lifestyle After Cancer survey (*N* = 588). Self-reported clinical, demographic, and physical activity (Godin Leisure-Time Exercise Questionnaire) data were collected. Logistic regression models assessed whether clinical and demographic factors were associated with meeting the MVPA guideline.

**Results:**

Most (59%) individuals with metastatic cancer were not meeting the WHO guideline of 150 min of MVPA per week. Adjusting for cancer type, the odds of meeting the MVPA guideline were lower for unemployed individuals (OR = 0.47, 95% CI = 0.23–0.77) and ethnic minorities (OR = 0.44, 95% CI = 0.22–0.89), but higher for those with a university education (OR = 1.89*, 95%* CI = 1.0–3.57). Most (63.5%) participants felt they should be doing more physical activity. However, 70.1% did not receive any physical activity advice or support, despite 73.6% wanting to receive it.

**Conclusion:**

People with metastatic cancer may need further support to address inadequate levels of physical activity. The differences observed between demographic sub-groups suggest this may be more beneficial for those less likely to engage in physical activity.

**Implications for Cancer Survivors:**

Individuals with metastatic cancer are likely to benefit from increased physical activity support, which considers the needs of diverse demographic groups.

**Supplementary Information:**

The online version contains supplementary material available at 10.1007/s11764-024-01700-5.

## Introduction

Around 20% of all cancer cases in the UK are diagnosed at the metastatic stage [1]. Improvements in treatment mean many individuals with metastatic cancer now live several years with the disease. However, compared with those diagnosed at earlier stages, people with metastatic cancer experience greater disease- and treatment-related challenges with their physical and psychological health [2–5]. As life expectancy increases, there is a need to improve the quality of life in individuals living with metastatic cancer.

Physical activity (PA) could be an effective and affordable intervention for improving symptom management and quality of life for those with metastatic disease. Recent evidence suggests PA can reduce fatigue and improve muscle function, quality of sleep, patient autonomy, and psychological and social function for individuals with metastatic cancer [6–10]. Moderate-vigorous physical activity (MVPA), is activity that causes an increase in heart and breathing rate [11], and may be associated with slower disease progression [12, 13] and improved survival [14, 15]. Systematic reviews of randomised controlled trials (RCTs) show PA is safe and feasible for this patient group, including those with bone metastases when supported by a trained professional [16–19].

Existing studies have concluded levels of PA in people with metastatic cancer are low [20–22]. However, an integrative review including 18 studies in metastatic breast cancer highlighted that most of this literature relies on small, convenience samples (*n* = 20–231), with 39% of included studies given a poor quality rating [22]. We identified one large-scale US study which compared PA levels among metastatic and non-metastatic patients with colorectal cancer (*n* = 875) [23]. They reported median level of PA was significantly lower in the metastatic group (3.4 metabolic equivalent task hours per week), compared with the non-metastatic group (4.6 h). A recent European study (Netherlands, Spain, Germany, Poland, Sweden) of individuals with metastatic breast cancer (*n* = 420), showed patients self-reported a median of 33 (IQR: 0–120) and 0 (IQR: 0–45) minutes of moderate and vigorous intensity PA per week, respectively. This is significantly below the World Health Organisation (WHO) recommendation of 150 min of MVPA per week for adults with cancer [24].

Observational and qualitative studies suggest patients experience a range of physical, psychological and logistical barriers to keeping active and adhering to PA programs [22, 25, 26]. Understanding the factors associated with PA in this group could help tailor and improve the effectiveness of future interventions [27]. In early-stage cancer survivors, individuals from lower income, ethnic minority, and non-University educated backgrounds, those affected by overweight or who have a high number of comorbidities are less likely to be active [28–31]. Some small studies have explored demographic and clinical factors associated with PA in metastatic cancer populations, but the findings are inconsistent. Two studies (*n* = 24; *n* = 141) showed Body Max Index (BMI) was not associated with PA [32, 33], but another (*n* = 50) showed being under- or of normal weight was associated with higher PA [34]. Similarly one study (*n* = 63) showed older patients were more likely to adhere to a PA intervention [35], but others showed no significant relationships between age [33, 36, 37], or other demographic groups and levels of PA [10, 38].

To inform the development of inclusive and effective interventions, there is a need to understand patient attitudes to PA and receiving support. This can help researchers tailor interventions to an individual’s interests, or align them to perceived physical capabilities, which can influence engagement [6, 39]. People with advanced cancer often report a reduction in PA from before their diagnosis [40–42] and express an interest in increasing it after [35, 39, 43, 44]. Similarly, qualitative studies suggest strong interest in receiving PA advice and support from healthcare providers, as access to this or appropriate PA programs appears limited [39, 41, 42, 45]. However, interest levels and willingness to participate in PA may vary across groups. For example, one cross-European study showed Swedish participants with metastatic breast cancer, had more positive attitudes towards exercising compared with Polish participants [39]. This study did not assess the attitudes of UK participants with metastatic cancer. Differences may also exist for patients with different types of metastatic cancer, as there are unique challenges associated with each group. No study has compared perceptions across disease sites. Additionally, it is not clear how common these perceptions are across the metastatic cancer population, as large-scale quantitative studies are lacking.

Inconsistencies in barriers and preferences may result from the varied needs of people with advanced cancer, and a reliance on small convenience samples. Larger studies are required to build on existing data. Using data from the “Health and Lifestyle After Cancer” cohort study [46], we aimed to estimate for the first time, how many UK individuals with metastatic breast, prostate, and colorectal cancer are meeting the WHO moderate-vigorous PA guideline, and what clinical and demographic factors predict the likelihood of meeting this guideline. Additionally, we aimed to explore patient perceptions about their pre-diagnosis and current PA, and whether they had received, or were interested in receiving PA advice, overall and between disease sites.

## Methods

Cross-sectional data from the "Health and Lifestyle After Cancer” survey were analysed [46]. Ethical approval was obtained through the National Research Ethics Service Committee South Central (Oxford B [14/SC/1369]).

### Sample

In ten National Health Service (NHS) sites in London and Essex, research nurses sent surveys to 13,645 adults diagnosed with breast, prostate, or colorectal cancer between 2012–2015. Patients were identified by research nurses, and consequently, the final sample included some patients diagnosed outside of these dates (range of most recent cancer diagnosis: 2001–2017). The response rate was 43% (*N* = 5,835). Participants were asked to report their most recent cancer type from breast, prostate or colorectal and were asked ‘Has this cancer spread to any other parts of your body?’ (“Yes”, “No”, “I don’t know” [coded as missing]). The present analysis was restricted to adults who selected yes to this question.

### Measures

#### Moderate-Vigorous Physical Activity (MVPA)

Participants reported how many times and for how many hours/minutes per week over the last month, they spent engaging in sessions of 15 min or more moderate (not exhausting) and strenuous (heart beats rapidly) physical activity in their free time. This measure was adapted from the validated Godin Leisure-Time Exercise Questionnaire (LTEQ; [47]) which only asked about number of 15-min or more sessions of exercise per week, not the duration. The LTEQ also asked questions about mild PA (minimal effort) but this item was not used for the calculation of MVPA. The number of strenuous activity minutes was doubled and added to the number of moderate activity minutes to calculate an MVPA composite, measured in minutes per week. MVPA was coded into “meeting guideline” and “not meeting guideline” based on the WHO guideline of 150 min of MVPA per week [24].

#### Physical activity perceptions

Participants reported which statement best described them at the time they completed the survey: “I think I should be doing more physical activity”, “I think I should be doing less physical activity”, “I don’t think I need to change my physical activity” or “Don’t know” (coded as missing). To assess perceived physical activity change since diagnosis, participants reported whether the amount of physical activity they do now was “more”, “about the same”, or “less” than before their diagnosis.

#### Physical activity advice

Participants indicated whether they had received advice from a health care professional to increase their physical activity (“yes” or “no”), and also rated their interest in receiving physical activity information or advice on a 4-point scale from “*not at all interested”,* to “*extremely interested*”.

#### Clinical factors

BMI was calculated from participant reported height and weight (kg/m^2^). Based on WHO guidelines (WHO, 1995), participants were categorised as “*underweight*” (BMI < 18.5), “*healthy weight*” (BMI ≥ 18.5 and < 25), “*overweight*” (BMI ≥ 25 and < 30), or “*obese*” (BMI ≥ 30). Total number of comorbidities was calculated based on the number of other health problems participants selected from a list of 15 conditions (e.g., diabetes, arthritis, angina, asthma, psychiatric illness), and providing details of any other condition. For type of treatments, participants selected what treatments they had previously received (surgery, radiotherapy, chemotherapy, hormone therapy, active surveillance, biological therapy), and provided details of any other treatment.. Finally, participants indicated the time since completing their main treatment which was coded as “s*till having main treatment”,* “*less than 1 year*”, “*more than 1 year*” or “*on active surveillance*”.

#### Demographic factors

Participants reported their current employment status and responses were recoded into: “w*orking*” (Employed full-time, employed part-time, self-employed, voluntary work, studying), “n*ot working*” (unemployed, retired, unable to work). Age was dichotomised based on the UK state pension age, into “*below retirement age”* (< 67 years) and “*above retirement age*” (≥ 67 years). For ethnicity, participants reported which ethnic group best described them from a list of options or specified another ethnic group. Due to there being small numbers of respondents for some of the ethnic minority groups, responses were recoded into “w*hite*” (1) and “n*on-white*” (2). Participants selected what educational or professional qualifications they had obtained from a list of options (including “*no formal qualification*”) or specified another qualification. Responses were recoded into an ordinal scale, from “*none*”, “*GCSE/vocational*”, “*A-level*” or “d*egree or above*”.

### Analysis

The analysis protocol was preregistered on the Open Science Framework prior to data analysis (https://osf.io/m7ktq/). All analyses were conducted in R [48].

Descriptive statistics were summarised for all variables. The frequency (%) of meeting the MVPA guideline was described, overall and across cancer type, and by clinical and demographic factors. Frequencies (%) of all measures of physical activity perceptions and advice, overall and across cancer type were also given. Although not pre-registered, we also conducted Chi-square tests to test if there were statistically significant differences in the frequency of meeting the MVPA guideline between the groups.

To assess whether clinical and demographic factors are associated with the probability an individual is meeting the MVPA guideline, logistic regression models were fitted. Models were adjusted for disease site. Potential predictors (BMI, comorbidities, type of treatments received, time since treatment, current employment, highest education, ethnicity, received physical activity advice, disease site) were added into the model in one step. Odds ratios and 95% confidence intervals were reported. For ordinal predictor variables (BMI, time since treatment, education), reference categories were chosen that would provide logical contrasts that we were interested in interpreting. For binary variables coded as 0 or 1 (treatment type, employment, ethnicity), level 0 was chosen as the default. For nominal, non-binary variables (cancer type), we chose the group that was the largest.

A missing data analysis ascertained 8.5% of values were missing overall, with Little’s Missing Completely at Random (MCAR) test suggesting the data was missing at random (*p* = 0.767). To address this, multiple imputation was used, with the R package *mice* [49]. A total of 35 imputations were conducted, which was based on the proportion of missing values [50, 51] and on convergence with a second imputed dataset.

In an exploratory analysis, we re-ran the logistic regression analysis while stratifying for disease site. This allowed us to explore whether the association between clinical and demographic factors, and the probability an individual is meeting the MVPA guideline, differs by type of primary cancer. Within prostate and colorectal cancer, the numbers of individuals receiving biological and hormone treatment were low (< 2) thus before analysis, we combined the five individual treatment variables (surgery, chemotherapy, radiotherapy, hormone treatment, biological therapy) to use the number of treatments received. No individuals with prostate cancer were classed as underweight for the BMI variable, so underweight and healthy weight were merged into one category (0), keeping the overweight (1) and obese (2) categories as they were specified.

## Results

The sample (*N* = 588/5835 = 10%) is described in Table 1. Breast cancer was the most common cancer type (45.2%), and there was a slightly higher percentage of females (56.7%). The mean age was 63.8 years, and the majority were not working (65.8%) and white (89.9%).

**Table 1:** Descriptive statistics and moderate-vigorous physical activity levels

		Total (*n* = *558*)			Meeting guideline (*n* = *167*)		Not meeting guideline (*n* = *242*)		Chi-squared test		
		Mean (SD)	*N*	%	*N*	%*	*N*	%	X^2^	df	*p*
Cancer type	Breast		252	45.2	91	43.8	117	56.3			
	Prostate		155	27.8	33	31.1	73	68.9			
	Colorectal		151	27.1	43	45.3	52	54.7			
									5.633	2	0.060
Sex	Male		241	43.3	57	36.3	100	63.7			
	Female		316	56.7	109	43.4	142	56.6			
									1.745	1	**0.019**
Age		63.8 (12.5)									
	Below retirement		309	55.7	125	49.0	130	51.0			
	Above retirement		246	44.3	40	26.3	112	73.7			
									19.434	1	** < 0.01**
Employment	Working		189	34.2	87	56.1	68	43.9			
	Not working		363	65.8	78	31.2	172	68.8			
									23.607	1	** < 0.001**
Highest level of education	None		133	26.7	17	24.3	53	75.7			
	GCSE/vocational		135	27.1	44	44.9	54	55.1			
	A-level		64	12.8	18	31.0	40	69.0			
	Degree or above		167	33.5	76	51.7	71	48.3			
									17.92	3	** < 0.001**
Ethnicity	White		499	89.9	153	42.7	205	57.3			
	Ethnic minority		56	10.1	13	27.1	35	72.9			
									3.668	1	0.055
BMI (kg/m^2^)	Underweight (BMI < 18.5)		5	0.9	2	66.7	1	33.3			
	Healthy weight (BMI ≥ 18.5 & < 25)		187	35.0	56	41.2	80	58.8			
	Overweight (BMI ≥ 25 & < 30)		224	42.0	75	43.4	98	56.7			
	Obese ≥ 30		118	22.1	27	32.9	55	67.1			
									-**	-	-
Time since treatment	Still having main treatment		167	30.7	35	30.7	79	69.3			
	Less than 1 year		73	13.4	21	42.9	28	57.1			
	More than 1 year		276	50.7	103	47.3	115	52.8			
	Active surveillance		28	5.2	6	30.0	14	70.0	9.572	3	**0.023**
Treatment received	Surgery		357	65.6	125	44.8	154	55.2	4.577	1	**0.033**
	Radiotherapy		320	58.6	104	41.8	145	58.2	0.020	1	0.887
	Chemotherapy		374	68.8	116	41.4	164	58.6	0.0841	1	0.772
	Biological therapy		14	2.6	4	33.3	8	66.7	-	-	-
	Hormone therapy		251	46.4	83	41.5	117	58.5	0	1	1
Comorbidities		1.25 (1.29)									
	0		178	31.9	70	50.7	68	49.3			
	1		197	35.5	56	38.4	90	61.6			
	2		108	19.4	27	36.0	48	64.0			
	≥ 3		75	13.4	14	28.0	36	72.0	10.093	3	**0.018**

*Proportion across each variable

**Chi-square tests could not be conducted for the BMI and Biological Therapy variables as there were fewer than 5 observations in at least one of the groups

Note: MVPA data was missing from 149/558 (26.7%) survey respondents

### Moderate-vigorous physical activity levels

Only 40.9% of individuals with metastatic cancer were meeting the WHO guideline of 150 min of weekly MVPA (Table 1). Those with metastatic prostate cancer were least likely to meet the guideline (31.1% meeting guideline [prostate], 43.8% [breast] and 45.3% [colorectal]). In our study, participants above retirement age (below retirement = 49% [meeting guideline] vs above retirement = 26.3%), those who were male (female = 43.4% vs male (36.3%), not working (working = 56.1% vs not working = 31.2%), with no educational qualifications (university degree or above = 51.7% vs no qualifications = 24.3%), and those with a greater number of comorbidities (0 comorbidities = 50.7% vs ≥ 3 comorbidities = 28%) were significantly less likely to meet the MVPA guideline.

In the multiple logistic regression analysis, the odds of meeting the MVPA guideline were lower for unemployed individuals, compared to employed individuals (OR = *0.49*; 95% CI = *0.30–0.79*) (Table 2). The odds were also lower for ethnic minorities than for white individuals (OR = *0.43*; 95% CI = *0.21–0.87*). Compared with those with no qualifications, individuals with a university education had greater odds of meeting the MVPA guideline (OR = *1.89, 95%* CI = *1.0–3.57*)*.*

**Table 2:** Logistic regression coefficients and odds ratios for meeting the moderate-vigorous physical activity guideline

		Coefficient	Std error	*p*	OR (95% CI)
Cancer type (ref: Breast)	Prostate	−0.13	0.41	0.744	0.88 (0.39–1.96)
	Colorectal	0.49	0.36	0.174	1.63 (0.80–3.33)
BMI (ref: Healthy weight)	Underweight	1.28	1.14	0.261	3.61 (0.38–34.06)
	Overweight	0.08	0.25	0.731	1.09 (0.66–1.80)
	Obese	−0.27	0.29	0.366	0.77 (0.43–1.37)
Comorbidities	Number of Comorbidities	−0.12	0.09	0.22	0.89 (0.74–1.07)
Time since treatment	Less than 1 year	0.42	0.37	0.253	1.52 (0.74–3.12)
(ref: Still having treatment)	More than 1 year	0.44	0.28	0.121	1.56 (0.89–2.72)
	Active surveillance	−0.04	0.54	0.937	0.96 (0.33–2.79)
Treatment (ref: Did not receive)	Surgery	0.07	0.34	0.834	1.07 (0.55–2.08)
	Radiotherapy	−0.12	0.27	0.664	0.89 (0.52–1.52)
	Chemotherapy	−0.41	0.30	0.172	0.66 (0.37–1.20)
	Hormone Therapy	0.39	0.28	0.163	1.47 (0.85–2.55)
	Biological therapy	−0.50	0.68	0.468	0.61 (0.16–2.34)
Employment (ref: Working)	Not working	−0.75**	0.24	0.002	0.47 (0.29–0.77)
Education (ref: No Qualifications)	GCSE/Vocational	0.38	0.33	0.251	1.47 (0.76–2.82)
	A level	0.03	0.39	0.944	1.03 (0.48–2.21)
	University education	0.63	0.33	0.055	1.88 (0.99–3.56)
Ethnicity (ref: White)	Ethnic Minority	−0.83*	0.36	0.023	0.44 (0.22–0.89)

*ref*: Reference category, *95% CI*: Lower – upper 95% confidence intervals. *Significant at *p* < 0.05; **Significant at *p* < 0.01

We ran the logistic regression while stratifying for disease site to test whether these findings varied by cancer site (Online Resource 1). After stratifying, having a university education no longer predicted the odds of meeting the MVPA guideline for breast, prostate or colorectal cancer. The odds of meeting the MVPA guideline were lower for unemployed individuals compared with employed individuals among the breast cancer (OR = *0.44, 95%* CI = *0.23–0.81*) and prostate cancer (OR = *0.19, 95%* CI = *0.06- 0.65)* subgroups. The odds were lower for ethnic minorities compared with white individuals (OR = *0.21, 95%* CI = *0.07–0.59)* for breast cancer only. No variables significantly predicted the odds of meeting the guideline for individuals with colorectal cancer.

### Perceptions and advice relating to physical activity

Nearly two thirds (63.5%) of participants felt they should be doing more PA, with 1.6% believing they should be doing less. Individuals with metastatic breast cancer were more likely to feel they should be doing more PA (71.1%) compared with those with metastatic prostate (57.9%) and colorectal cancer (55.6%). Over half (53.3%) of participants believed they were less active now compared with before their cancer diagnosis. Only 12.5% believed they were more active. Percentages were similar across the three cancer types (Table 3).

**Table 3:** Perceptions of physical activity overall and by cancer type

		Total		Breast		Prostate		Colorectal	
		*N*	%	*N*	%	*N*	%	*N*	%
Present physical activity	Should be doing more	309	63.45		71.1	77	57.9	70	55.6
	Should be doing less	8	1.64		0.4	3	2.3	4	3.2
	Don't need to change	170	34.91	162	28.5	53	39.9	52	41.3
	N	487		1		133		126	
Physical activity change	More	69	12.5	45	17.9	12	7.9	12	8.1
	About the same	189	34.2	77	30.7	58	38.2	54	36.2
	Less	294	53.3	129	51.4	82	54.0	83	55.7
	N	552		251		152		149	

The majority (70.1%) of participants reported they did not receive any advice or support from a healthcare provider related to PA. This was slightly higher among those with colorectal cancer (75.2%) compared with breast (66.7%) and prostate (71.2%). Overall, 73.6% of participants expressed some interest in receiving advice to help increase their PA. Interest was generally higher among those with breast cancer with 79.4% overall reporting some interest. Interest was lowest among those with colorectal cancer, with 38% reporting they were not interested in receiving any advice (Table 4).

**Table 4:** Physical activity advice received and interest overall and by cancer type

		Total		Breast		Prostate		Colorectal	
		*N*	%	*N*	%	*N*	%	*N*	%
PA advice received	Yes	155	29.9	80	33.3	42	28.8	33	24.8
	No	364	70.1	160	66.7	104	71.2	100	75.2
	N	519		240		146		133	
Interest in PA advice	Not interested	127	26.5	48	20.6	33	26.2	46	38.0
	A little interested	79	16.5	38	16.3	25	19.8	16	13.2
	Somewhat interested	116	24.2	51	21.9	37	29.4	28	23.1
	Very interested	158	32.9	96	41.2	31	24.6	31	25.6
	N	480		233		126		121	

## Discussion

In this UK observational study, 59% of individuals living with metastatic breast, prostate, and colorectal cancer were not meeting the MVPA guideline. The majority felt they needed to be doing more PA and expressed an interest in receiving PA advice, however, 70% of participants had not received any since their diagnosis. We identified potential educational and ethnic disparities in meeting the MVPA guideline, which should be considered alongside attempts to resolve inequalities across the cancer spectrum. The low estimates of MVPA suggest PA support for individuals living with breast, prostate, and colorectal metastatic cancer is needed. Our findings strengthen the previous evidence base estimating insufficient levels of PA, in smaller or non-UK samples [23].

Providing PA support which considers the reduced physical function and psychological challenges could be helpful in increasing PA for this patient group, but availability of this support appears to be limited. The majority of our sample reported not having received any PA advice post-diagnosis. Individuals with metastatic cancer have previously reported feeling insecure about their physical limitations and unsafe performing exercises without supervision from a healthcare professional [40, 45]. Healthcare professionals themselves have indicated a need for further support in discussing PA recommendations for patients with metastatic cancer, given the complexities of the disease presentation [52, 53]. Despite perceiving PA as being beneficial, some physiotherapists have expressed uncertainty about prescribing it to metastatic patients with bone metastases, due to concerns about an increased risk of injury [52]. The International Bone Metastases Exercise Working Group (IBMEWG), an international and multidisciplinary group of expert clinicians and researchers in exercise oncology, recently released the first clinical exercise guidelines for people with bone metastases in 2022 [19, 54]. The IBMEWG was recently formed, with the group recognising the lack of clinical guidance around exercise for people with metastatic cancer and bone metastases means many healthcare providers are not providing advice, despite the benefit this could have. The development of these best practice recommendations are an integral step, but further efforts are needed to ensure healthcare professionals are given the appropriate support and training to facilitate their rapid implementation.

We also showed differences in the odds of meeting the MVPA guideline between different ethnicities, employment statuses, and education levels. Similar disparities have been shown in those with early-stage cancer, with those from ethnic minority backgrounds and lower socioeconomic status less likely to be active [29–31, 55]. Underserved or disadvantaged demographic groups are likely to experience greater barriers to being physically active, such as a lack of advice, availability of support programs, and financial support to attend programs [56–58]. A study with breast, prostate, and colorectal cancer survivors (*n* = 1299), which included both metastatic and non-metastatic patients, found participants with lower educational attainment and who were not employed reported a higher number of perceived structural barriers to PA [56]. Underserved demographic groups may need more, or different kinds of support, to increase their PA and researchers should actively involve these diverse communities when developing inclusive interventions.

PA support may be more beneficial if it is sensitive to the needs of different subgroups [22, 40, 42, 59, 60]. In addition to demographic differences, individuals with metastatic cancer are heterogeneous in terms of tumour type, treatment plans, disease progression, symptom type and severity, and other individual contexts [61, 62]. In qualitative studies, individuals with metastatic cancer highlight preferences for support tailored to their varied abilities and needs, and sensitive to how this changes throughout their disease [26, 42, 45]. Exercise programs that are based on the location of the bone metastases have been shown to be safe and efficacious for men with prostate cancer in small trials [63, 64]. Future research could explore further, cost-effective ways of tailoring PA for this population, for example by using adaptive intervention designs [65, 66].

After stratifying by disease site, the effect of highest education was no longer statistically significant for any of the cancer types, nor was ethnicity for individuals with colorectal cancer. This finding is likely to be explained by the smaller sample size for each patient group, and consequent lower statistical power to detect significant effects. However, ethnicity no longer predicted the odds of meeting the MVPA guideline for prostate and colorectal cancer, and employment was no longer a predictor for colorectal cancer. There were some further differences for individuals with metastatic prostate cancer; a smaller proportion of prostate cancer survivors were meeting the MVPA guideline, and they were slightly more likely to feel they should be doing more PA, compared to breast and colorectal survivors. To our knowledge, this study is the first to compare levels of MVPA between different metastatic cancer types. Some studies in early cancer survivors show similar patterns, with higher rates of PA in breast cancer survivors compared to prostate cancer [67]. However, others have found opposite findings, with breast cancer survivors engaging in less activity compared to those with prostate and colorectal cancer [68, 69]. Differences between breast and prostate cancer could be associated with gender differences, but it is unclear if gender differences explain the differences between disease sites, or vice versa. This suggests further research comparing across cancer types is required, as well as elucidating the role of gender within a metastatic clinical context to try and understand which groups may need more support.

### Limitations

Our study had limitations. We used self-reported scales to assess PA. While a validated scale was used to assess MVPA (LTEQ: [47]), individuals may have overestimated their PA compared with accelerometer-assessed activity. Previous evidence from cancer survivors suggests PA is lower when assessed with an accelerometer than self-reported data [70, 71]. Participants also self-reported whether they had metastatic cancer, which was based on disease spread. We may have incorrectly included or excluded members of the wider study sample of cancer survivors, which would affect the generalisability of the study findings to the metastatic population. However, previous studies suggest that many cancer survivors are not able to identify their cancer stage accurately [72, 73], therefore asking about spread, rather than cancer stage, may have helped us reach more eligible participants. Additionally, our sample was partly self-selecting, as completion of the survey was voluntary. It may be those who chose to complete a health behaviour survey were more likely to be active. Taken together, the proportion of individuals with metastatic breast, prostate, and colorectal cancer who are meeting PA guidelines may have been even lower than we estimate here. However, the question about MVPA asked about strenuous and moderate PA in the participants’ free time, thus better reflects planned exercise specifically, rather than any PA which may be integrated into work or daily life. Therefore, actual levels of MVPA could actually have been higher than reported. Our data were cross-sectional, and only associations with the likelihood of meeting the MVPA guideline were explored. Causality cannot be inferred. We also based our assessment of meeting the PA guideline on just the MVPA aspect of the WHO guidelines for people with chronic conditions, however there are other key aspects of PA that are reflected in these guidelines, such as muscle strengthening activities and functional balance training. Therefore, an individual participant may not have met the specific MVPA guideline but did meet another PA guideline, or vice versa, which was not reflected in our study. Finally, despite good representation of employment status, sex, and educational attainment, only 10% of our sample were from non-White backgrounds. The decision to dichotomise ethnicity could ignore real cultural differences between groups [30, 55, 74].

## Conclusion

Overall, our study provides further evidence that physical activity levels in the metastatic breast, prostate, and colorectal cancer population are low and suggest further support is needed. Our analyses showed the majority of individuals with metastatic breast, prostate, and colorectal cancer in our study were not meeting the MVPA guideline and felt they should be doing more activity. We also showed most participants had not received any support or advice from healthcare providers, despite expressing an interest in receiving this. The recent publication of the first clinical exercise guidelines for individuals with bone metastases could help improve this support, provided healthcare staff are sufficiently trained to facilitate the implementation of this guidance. However, the differences in the odds of meeting the MVPA guideline between different demographic groups suggest support may be more needed in certain groups and diversity should be considered when developing accessible and inclusive interventions.

## Supplementary Information

Below is the link to the electronic supplementary material.

Supplementary file1 (DOCX 42 KB)

## Data Availability

This study’s methods, analysis plan and hypotheses were pre-registered on the Open Science Framework prior to data analysis (https://osf.io/m7ktq/). Following publication, the study’s code will be uploaded to this OSF repository. The questionnaire and dataset used in this study is available on request. For the purpose of open access, the authors have applied a Creative Commons Attribution (CC BY) licence to any Author Accepted Manuscript version arising.

## References

[1] Cancer Research UK. Proportion of cancer cases by stage at diagnosis, England, 2018. Early Diagn. Data Hub. 2018. https://crukcancerintelligence.shinyapps.io/EarlyDiagnosis/. Accessed 10 Aug 2022.

[2] Cheville AL, Troxel AB, Basford JR, Kornblith AB. Prevalence and treatment patterns of physical impairments in patients with metastatic breast cancer. J Clin Oncol. 2008;26(16):2621–9. 10.1200/JCO.2007.12.3075. 18509174 10.1200/JCO.2007.12.3075 PMC4524509

[3] Dunn J, Watson M, Aitken JF, Hyde MK. Systematic review of psychosocial outcomes for patients with advanced melanoma. Psychooncology. 2017;26(11):1722–31. 10.1002/PON.4290. 27696578 10.1002/pon.4290

[4] Mollica MA, et al. Survivorship for individuals living with advanced and metastatic cancers: national cancer institute meeting report. JNCI J Natl Cancer Inst. 2022;114(4):489–95. 10.1093/jnci/djab223. 34878107 10.1093/jnci/djab223 PMC9002286

[5] Shallwani SM, Simmonds MJ, Kasymjanova G, Spahija J. Quality of life, symptom status and physical performance in patients with advanced non-small cell lung cancer undergoing chemotherapy: an exploratory analysis of secondary data. Lung Cancer. 2016;99:69–75. 10.1016/J.LUNGCAN.2016.06.018. 27565917 10.1016/j.lungcan.2016.06.018

[6] Albrecht TA, Taylor AG. Physical activity in patients with advanced-stage cancer: a systematic review of the literature. Clin J Oncol Nurs. 2012;16(3):293. 10.1188/12.CJON.293-300. 22641322 10.1188/12.CJON.293-300 PMC4408536

[7] Bobrowski D, et al. A review of the effect of exercise on patients with advanced cancer. J Pain Manage. 2018;11(3):267–85.

[8] Chen Y-j, et al. Exercise training for improving patient-reported outcomes in patients with advanced-stage cancer: a systematic review and meta-analysis. J Pain Symptom Manage. 2020;59(3):734-749.e10. 10.1016/J.JPAINSYMMAN.2019.09.010. 31546002 10.1016/j.jpainsymman.2019.09.010

[9] Rodríguez-Cañamero S, et al. Impact of physical exercise in advanced-stage cancer patients: systematic review and meta-analysis. Cancer Med. 2022;00:1–14. 10.1002/CAM4.4746. 10.1002/cam4.4746 PMC9554454 35411694

[10] Takemura N, Chan SL, Smith R, Cheung DST, Lin CC. The effects of physical activity on overall survival among advanced cancer patients: a systematic review and meta-analysis. BMC Cancer. 2021;21(1):1–13. 10.1186/S12885-021-07988-1/FIGURES/4. 33678180 10.1186/s12885-021-07988-1 PMC7938536

[11] Rennie KL, Hemingway H, Kumari M, Brunner E, Malik M, Marmot M. Effects of moderate and vigorous physical activity on heart rate variability in a british study of civil servants. Am J Epidemiol. 2003;158(2):135–43. 10.1093/AJE/KWG120. 12851226 10.1093/aje/kwg120

[12] Dai JY, et al. Vigorous physical activity is associated with lower risk of metastatic-lethal progression in prostate cancer and hypomethylation in the CRACR2A gene. Cancer Epidemiol Biomarkers Prev. 2019;28(2):258–64. 10.1158/1055-9965.EPI-18-0622. 30464020 10.1158/1055-9965.EPI-18-0622 PMC6363836

[13] Delrieu L, et al. Impact of physical activity on oxidative stress markers in patients with metastatic breast cancer. Oxid Med Cell Longev. 2021;2021:e6694594. 10.1155/2021/6694594. 10.1155/2021/6694594 PMC8302399 34326920

[14] Delrieu L, et al. Analysis of the StoRM cohort reveals physical activity to be associated with survival in metastatic breast cancer. Sci Rep 2020 101. 2020;10(1):1–12. 10.1038/s41598-020-67431-6. 10.1038/s41598-020-67431-6 PMC7329808 32612272

[15] Smit KC, et al. Physical activity is associated with improved overall survival among patients with metastatic colorectal cancer. Cancers. 2022;14(4):Art. no. 4. 10.3390/cancers14041001. 10.3390/cancers14041001 PMC8870120 35205748

[16] De Lazzari N, Niels T, Tewes M, Götte M. A systematic review of the safety, feasibility and benefits of exercise for patients with advanced cancer. Cancers 2021. 2021;13(17):4478. 10.3390/CANCERS13174478. 10.3390/cancers13174478 PMC8430671 34503288

[17] Heywood R, McCarthy AL, Skinner TL. Safety and feasibility of exercise interventions in patients with advanced cancer: a systematic review. Support Care Cancer. 2017;25(10):3031–50. 10.1007/S00520-017-3827-0/TABLES/4. 28741176 10.1007/s00520-017-3827-0

[18] Weller S, et al. Exercise for individuals with bone metastases: a systematic review. Crit Rev Oncol Hematol. 2021;166:103433. 10.1016/j.critrevonc.2021.103433. 34358650 10.1016/j.critrevonc.2021.103433

[19] Campbell KL, et al. Exercise recommendation for people with bone metastases: expert consensus for health care providers and exercise professionals. JCO Oncol Pract. 2022;18(5):e697–709. 10.1200/OP.21.00454. 34990293 10.1200/OP.21.00454 PMC9810134

[20] Zopf EM, et al. Associations between aerobic exercise levels and physical and mental health outcomes in men with bone metastatic prostate cancer: a cross-sectional investigation. Eur J Cancer Care (Engl). 2017;26(6):n/a-N.PAG. 10.1111/ecc.12575. 10.1111/ecc.12575 27647712

[21] Silva TD, Boing L, Dias M, Pazin J, Guimarães AC de A. Prostate cancer: quality of life and physical activity level of patients. J Phys Educ. 2018;29:e2932. 10.4025/jphyseduc.v29i1.2932.

[22] Geng Z, Wang J, Zhang Y, Wu F, Yuan C. Physical activity in the context of advanced breast cancer: an integrative review. J Adv Nurs. 2021;77(5):2119–43. 10.1111/JAN.14709. 33314310 10.1111/jan.14709

[23] Van Loon K, et al. Comparison of dietary and lifestyle habits among stage III and metastatic colorectal cancer patients: findings from CALGB 89803 and CALGB 80405. Clin Colorectal Cancer. 2013;12(2):95–102. 10.1016/J.CLCC.2012.11.002. 23317558 10.1016/j.clcc.2012.11.002 PMC3790266

[24] World Health Organization. Guidelines on physical activity and sedentary behavior. Geneva: World Health Organization; 2020.

[25] Clark MM, et al. Physical activity in patients with advanced-stage cancer actively receiving chemotherapy. J Support Oncol. 2007;5(10):487–93. 18240671

[26] ten Tusscher MR, et al. Physical problems, functional limitations, and preferences for physical therapist-guided exercise programs among Dutch patients with metastatic breast cancer: a mixed methods study. Support Care Cancer. 2019;27(8):3061–70. 10.1007/S00520-018-4619-X/TABLES/3. 30610432 10.1007/s00520-018-4619-x

[27] Coups EJ, et al. Correlates of physical activity among lung cancer survivors. Psychooncology. 2009;18(4):395–404. 10.1002/PON.1520. 19241488 10.1002/pon.1520 PMC2778598

[28] Speed-Andrews AE, et al. Medical, demographic and social cognitive correlates of physical activity in a population-based sample of colorectal cancer survivors. Eur J Cancer Care (Engl). 2012;21(2):187–96. 10.1111/J.1365-2354.2011.01290.X. 21902736 10.1111/j.1365-2354.2011.01290.x

[29] Hong S, et al. Correlates of physical activity level in breast cancer survivors participating in the Women’s Healthy Eating and Living (WHEL) Study. Breast Cancer Res Treat 2006 1012. 2006;101(2):225–32. 10.1007/S10549-006-9284-Y. 10.1007/s10549-006-9284-y 17028988

[30] Irwin ML, et al. Physical activity levels among breast cancer survivors. Med Sci Sports Exerc. 2004;36(9):1484. 10.1249/01.MSS.0000074670.03001.98. 15354027 PMC3000611

[31] Naik H, et al. Socioeconomic status and lifestyle behaviours in cancer survivors: smoking and physical activity. Curr Oncol. 2016;23(6):Art. no. 6. 10.3747/co.23.3166. 10.3747/co.23.3166 PMC5176380 28050143

[32] Lowe SS, Danielson B, Beaumont C, Watanabe SM, Baracos VE, Courneya KS. Correlates of objectively measured sedentary behavior in cancer patients with brain metastases: an application of the theory of planned behavior. Psychooncology. 2015;24(7):757–62. 10.1002/pon.3641. 25073628 10.1002/pon.3641

[33] Frikkel J, et al. Fatigue, barriers to physical activity and predictors for motivation to exercise in advanced cancer patients. BMC Palliat Care. 2020;19(1):43. 10.1186/s12904-020-00542-z. 32234027 10.1186/s12904-020-00542-z PMC7110817

[34] Lowe SS, Watanabe SM, Baracos VE, Courneya KS. Determinants of physical activity in palliative cancer patients: an application of the theory of planned behavior. J Support Oncol. 2011;10(1):30–6. 10.1016/j.suponc.2011.07.005. 21996569 10.1016/j.suponc.2011.07.005

[35] Oldervoll LM, et al. Are palliative cancer patients willing and able to participate in a physical exercise program? Palliat Support Care. 2005;3(4):281–7. 10.1017/S1478951505050443. 17039983 10.1017/s1478951505050443

[36] Schofield C, et al. Activity behaviors and physiological characteristics of women with advanced-stage ovarian cancer: a preliminary cross-sectional investigation. Int J Gynecol Cancer. 2018;28(3):604–13. 10.1097/IGC.0000000000001197. 29369120 10.1097/IGC.0000000000001197

[37] Ligibel JA, et al. Physical activity, weight, and outcomes in patients receiving chemotherapy for metastatic breast cancer (C40502/Alliance). JNCI Cancer Spectr. 2021;5(3). 10.1093/jncics/pkab025. 10.1093/jncics/pkab025 PMC8103727 33981951

[38] Norris RL, Liu Q, Bauer-Wu S. Age and functional ability are associated with self-care practices used by women with metastatic breast cancer: an exploratory study. J Nurs Healthc Chronic Illn. 2009;1(1):71–7. 10.1111/j.1365-2702.2008.01004.x.

[39] Sweegers MG, et al. Perspectives of patients with metastatic breast cancer on physical exercise programs: results from a survey in five European countries. Support Care Cancer. 2023;31(12):694. 10.1007/s00520-023-08124-4. 37955790 10.1007/s00520-023-08124-4 PMC10643348

[40] Mikkelsen MK, Nielsen DL, Vinther A, Lund CM, Jarden M. Attitudes towards physical activity and exercise in older patients with advanced cancer during oncological treatment – a qualitative interview study. Eur J Oncol Nurs. 2019;41:16–23. 10.1016/J.EJON.2019.04.005. 31358249 10.1016/j.ejon.2019.04.005

[41] Agnew M, et al. “There is no expiration date”: a qualitative analysis using the Social Cognitive Theory to identify factors influencing physical activity among adults living with advanced cancer. Support Care Cancer Off J Multinatl Assoc Support Care Cancer. 2024;32(4):242. 10.1007/s00520-024-08440-3. 10.1007/s00520-024-08440-3 PMC11373671 38514490

[42] Sheill G, Guinan E, Neill LO, Hevey D, Hussey J. The views of patients with metastatic prostate cancer towards physical activity: a qualitative exploration. Support Care Cancer. 2018;26(6):1747–54. 10.1007/S00520-017-4008-X/TABLES/3. 29243168 10.1007/s00520-017-4008-x

[43] Knowlton SE, et al. Moving forward on all fronts: impact, patterns, and barriers to exercise in cancer survivors and patients living with advanced disease. Support Care Cancer. 2020;28(10):4979–88. 10.1007/s00520-020-05344-w. 32034513 10.1007/s00520-020-05344-w

[44] Lowe SS, Watanabe SM, Baracos VE, Courneya KS. Physical activity interests and preferences in palliative cancer patients. Support Care Cancer Off J Multinatl Assoc Support Care Cancer. 2010;18(11):1469–75. 10.1007/s00520-009-0770-8. 10.1007/s00520-009-0770-8 19902273

[45] Depenbusch J, et al. PERSPECTIVEs on supervised exercise programs in people with metastatic breast cancer- a qualitative study in four European countries. Support Care Cancer. 2023;31(5):281. 10.1007/s00520-023-07739-x. 37074497 10.1007/s00520-023-07739-x PMC10115708

[46] Beeken RJ, et al. Study protocol for a randomised controlled trial of brief, habit-based, lifestyle advice for cancer survivors: exploring behavioural outcomes for the Advancing Survivorship Cancer Outcomes Trial (ASCOT). Br Med J. 2016;6(11):e011646. 10.1136/bmjopen-2016-011646. 10.1136/bmjopen-2016-011646 PMC5178807 27881518

[47] Godin G, Shephard RJ. A simple method to assess exercise behavior in the community. Can J Appl Sport Sci. 1985;10(3):141–146. https://europepmc.org/article/med/4053261. Accessed 29 Aug 2022. 4053261

[48] R Core Team. R: a language and environment for statistical computing. Vienna: R Foundation for Statistical Computing; 2023. https://www.R-project.org/. Accessed 7 Feb 2023.

[49] van Buuren S, Groothuis-Oudshoorn K. mice: multivariate imputation by chained equations in R. J Stat Softw. 2011;45(3):1–67. 10.18637/JSS.V045.I03.

[50] Lee KJ, Roberts G, Doyle LW, Anderson PJ, Carlin JB. Multiple imputation for missing data in a longitudinal cohort study: a tutorial based on a detailed case study involving imputation of missing outcome data. Int J Soc Res Methodol. 2016;19(5):575–91. 10.1080/13645579.2015.1126486.

[51] White IR, Royston P, Wood AM. Multiple imputation using chained equations: issues and guidance for practice. Stat Med. 2011;30(4):377–99. 10.1002/sim.4067. 21225900 10.1002/sim.4067

[52] Sheill G, Guinan E, Neill LO, Hevey D, Hussey J. Physical activity and advanced cancer: the views of oncology and palliative care physicians in Ireland. Ir J Med Sci 1971 -. 2018;187(2):337–42. 10.1007/s11845-017-1677-x. 10.1007/s11845-017-1677-x 28861844

[53] Ten Tusscher MR, Groen WG, Geleijn E, Berkelaar D, Aaronson NK, Stuiver MM. Education needs of dutch physical therapists for the treatment of patients with advanced cancer: a mixed methods study. Phys Ther. 2020;100(3):477–86. 10.1093/ptj/pzz172. 32031218 10.1093/ptj/pzz172

[54] Hart NH, et al. Exercise for people with bone metastases: MASCC endorsed clinical recommendations developed by the International Bone Metastases Exercise Working Group. Support Care Cancer. 2022;30(9):7061–5. 10.1007/s00520-022-07212-1. 35710641 10.1007/s00520-022-07212-1

[55] Glenn BA, et al. Obesity, physical activity, and dietary behaviors in an ethnically-diverse sample of cancer survivors with early onset disease. J Psychosoc Oncol. 2018;36(4):418–36. 10.1080/07347332.2018.1448031. 29764334 10.1080/07347332.2018.1448031 PMC6209096

[56] Depenbusch J, et al. Impact and determinants of structural barriers on physical activity in people with cancer. Int J Behav Med. 2022;29(3):308–20. 10.1007/s12529-021-10014-0. 34550527 10.1007/s12529-021-10014-0 PMC9166881

[57] Powell L, Slater S, Chaloupka F. The relationship between community physical activity settings and race, ethnicity and socioeconomic status. Evid-Based Prev Med. 2004;1(2):135–44.

[58] Sequeira S, Cruz C, Pinto D, Santos L, Marques A. Prevalence of barriers for physical activity in adults according to gender and socioeconomic status. Br J Sports Med. 2011;45(15):A18–9. 10.1136/bjsports-2011-090606.59.

[59] Cheung WY, Le LW, Gagliese L, Zimmermann C. Age and gender differences in symptom intensity and symptom clusters among patients with metastatic cancer. Support Care Cancer. 2011;19(3):417–23. 10.1007/S00520-010-0865-2/TABLES/4. 20333411 10.1007/s00520-010-0865-2

[60] Wilk M, Kepski J, Kepska J, Casselli S, Szmit S. Exercise interventions in metastatic cancer disease: a literature review and a brief discussion on current and future perspectives. BMJ Support Palliat Care. 2020;10:404–10. 10.1136/bmjspcare-2020-002487. 32943468 10.1136/bmjspcare-2020-002487

[61] Krigel S, Myers J, Befort C, Krebill H, Klemp J. “Cancer changes everything!” Exploring the lived experiences of women with metastatic breast cancer. Int J Palliat Nurs. 2014;20(7):334–42. 10.12968/ijpn.2014.20.7.334. 25062379 10.12968/ijpn.2014.20.7.334

[62] Willis K, Lewis S, Ng F, Wilson L. The experience of living with metastatic breast cancer—a review of the literature. Health Care Women Int. 2015;36(5):514–42. 10.1080/07399332.2014.896364. 24579717 10.1080/07399332.2014.896364

[63] Cormie P, Newton RU, Spry N, Joseph D, Taaffe DR, Galvão DA. Safety and efficacy of resistance exercise in prostate cancer patients with bone metastases. Prostate Cancer Prostatic Dis. 2013;16(4):Art. no. 4. 10.1038/pcan.2013.22. 10.1038/pcan.2013.22 23917308

[64] Galvão DA, et al. Exercise preserves physical function in prostate cancer patients with bone metastases. Med Sci Sports Exerc. 2018;50(3):393. 10.1249/MSS.0000000000001454. 29036016 10.1249/MSS.0000000000001454 PMC5828380

[65] Hardeman W, Houghton J, Lane K, Jones A, Naughton F. A systematic review of just-in-time adaptive interventions (JITAIs) to promote physical activity. Int J Behav Nutr Phys Act. 2019;16(1):1–21. 10.1186/S12966-019-0792-7/TABLES/5. 30943983 10.1186/s12966-019-0792-7 PMC6448257

[66] Lei H, Nahum-Shani I, Lynch K, Oslin D, Murphy SA. A “SMART” design for building individualized treatment sequences. Annu Rev Clin Psychol. 2012;8(1):21–48. 10.1146/annurev-clinpsy-032511-143152. 22224838 10.1146/annurev-clinpsy-032511-143152 PMC3887122

[67] Steindorf K, et al. Change patterns and determinants of physical activity differ between breast, prostate, and colorectal cancer patients. Support Care Cancer. 2020;28(7):3207–18. 10.1007/s00520-019-05097-1. 31720802 10.1007/s00520-019-05097-1

[68] Blanchard CM, Stein K, Courneya KS. Body mass index, physical activity, and health-related quality of life in cancer survivors. Med Sci Sports Exerc. 2010;42(4):665–71. 10.1249/mss.0b013e3181bdc685. 19952838 10.1249/MSS.0b013e3181bdc685

[69] LeMasters TJ, Madhavan SS, Sambamoorthi U, Kurian S. Health behaviors among breast, prostate, and colorectal cancer survivors: a US population-based case-control study, with comparisons by cancer type and gender. J Cancer Surviv. 2014;8(3):336–48. 10.1007/s11764-014-0347-5. 24532045 10.1007/s11764-014-0347-5 PMC4892177

[70] Boyle T, Lynch BM, Courneya KS, Vallance JK. Agreement between accelerometer-assessed and self-reported physical activity and sedentary time in colon cancer survivors. Support Care Cancer. 2015;23(4):1121–6. 10.1007/S00520-014-2453-3/TABLES/2. 25301224 10.1007/s00520-014-2453-3

[71] Lally P, Miller NE, Lawrence C, Beeken RJ, Fisher A. Associations of self-reported and device-assessed physical activity with fatigue, quality of life, and sleep quality in adults living with and beyond cancer. J Sport Health Sci. 2023;12(6):664–73. 10.1016/j.jshs.2023.05.001. 37172763 10.1016/j.jshs.2023.05.001 PMC10658319

[72] Hinchey J, Goldberg J, Linsky S, Linsky R, Jeon S, Schulman-Green D. Knowledge of cancer stage among women with nonmetastatic breast cancer. J Palliat Med. 2016;19(3):314–7. 10.1089/jpm.2015.0133. 26855201 10.1089/jpm.2015.0133 PMC8109048

[73] Santoso JT, Engle DB, Schaffer L, Wan JY. Cancer diagnosis and treatment: communication accuracy between patients and their physicians. Cancer J Sudbury Mass. 2006;12(1):73–6. 10.1097/00130404-200601000-00013. 10.1097/00130404-200601000-00013 16613666

[74] Bryan SN, Tremblay MS, Pérez CE, Ardern CI, Katzmarzyk PT. Physical activity and ethnicity. Can J Public Health 2006 974. 2006;97(4):271–6. 10.1007/BF03405602. 10.1007/BF03405602 PMC6976107 16967744

