# Supplementary material for: Factors associated with physical activity in individuals with metastatic cancer: a UK cross-sectional survey
Source: J Cancer Surviv. 2024 Oct 28;20(3):967–77. doi: 10.1007/s11764-024-01700-5 (PMC13144276; doi:10.1007/s11764-024-01700-5)
Supplement: Supplementary file 1 — Supplementary file1 (DOCX 42 KB) [file 11764_2024_1700_MOESM1_ESM.docx]

**Online Resources (Supplementary Materials)**

**Factors associated with physical activity in individuals with metastatic cancer: A UK cross-sectional survey.**

**Journal of Cancer Survivorship**

**Authors:** Zainab Faatimah Haider^1^, Samuel George Smith^1^, Rebecca Walwyn^2^, Phillippa Lally^3^ & Abigail Fisher^4^, Rebecca Beeken^1^

**Affiliations:**

**^1^**Leeds Institute of Health Sciences, University of Leeds, Leeds, UK.

^2^Leeds Institute of Clinical Trials Research, University of Leeds, Leeds, UK

^3^University of Surrey, Surrey, UK

^4^University College London, Institute of Epidemiology and Health Care, London, UK

**Corresponding author:** Zainab Faatimah Haider

Email: [ll16zfh@leeds.ac.uk](mailto:ll16zfh@leeds.ac.uk)

Work Telephone: +441133431049

**Online Resource 1**

***Table S1: Logistic Regression Coefficients and Odds Ratios for Meeting MVPA Guidelines Stratified by Cancer Type (Breast Cancer)***

|  |  |  | **Coefficient** | **Std Error** | ***p*** | **OR** | **LC95%** | **UC95%** |
| --- | --- | --- | --- | --- | --- | --- | --- | --- |
| **BMI (ref: Underweight/healthy weight)** |  | Overweight | -0.441 | 0.364 | 0.228 | 0.643 | 0.313 | 1.322 |
|  |  | Obese | -0.620 | 0.417 | 0.139 | 0.538 | 0.236 | 1.226 |
|  |  |  |  |  |  |  |  |  |
| **Comorbidities** |  | Number of Comorbidities | -0.214 | 0.136 | 0.118 | 0.808 | 0.618 | 1.056 |
|  |  |  |  |  |  |  |  |  |
| **Time since treatment** |  | Less than 1 year | 0.546 | 0.656 | 0.407 | 1.726 | 0.472 | 6.315 |
| **(ref: Still having treatment)** |  | More than 1 year | 0.446 | 0.457 | 0.332 | 1.562 | 0.632 | 3.861 |
|  |  | Active surveillance | 0.698 | 1.019 | 0.494 | 2.010 | 0.268 | 15.083 |
|  |  |  |  |  |  |  |  |  |
| **Treatment** |  | Number of Treatments | -0.036 | 0.211 | 0.867 | 0.965 | 0.635 | 1.467 |
|  |  |  |  |  |  |  |  |  |
| **Employment (ref: Working)** |  | Not working | -0.846 | 0.322 | 0.009 | 0.429 | 0.227 | 0.810 |
|  |  |  |  |  |  |  |  |  |
| **Education (ref: No Qualifications)** |  | GCSE/Vocational | 0.172 | 0.579 | 0.767 | 1.187 | 0.376 | 3.745 |
|  |  | A level | 0.639 | 0.657 | 0.332 | 1.895 | 0.516 | 6.960 |
|  |  | University education | 0.829 | 0.571 | 0.150 | 2.290 | 0.738 | 7.107 |
|  |  |  |  |  |  |  |  |  |
| **Ethnicity (ref: White)** |  | Ethnic Minority | -1.580** | 0.537 | **0.004** | 0.206 | 0.071 | 0.593 |

Note: ref = Reference category. *Significant at *p<.05;* **Significant at *p<.01*

***Table S2: Logistic Regression Coefficients and Odds Ratios for Meeting MVPA Guideline Stratified by Cancer Type (Prostate Cancer)***

|  |  |  | **Coefficient** | **Std Error** | ***p*** | **OR** | **LC95%** | **UC95%** |
| --- | --- | --- | --- | --- | --- | --- | --- | --- |
| **BMI (ref: Underweight/healthy weight)** |  | Overweight | 0.353 | 0.513 | 0.493 | 1.423 | 0.514 | 3.941 |
|  |  | Obese | -0.203 | 0.691 | 0.770 | 0.816 | 0.206 | 3.235 |
|  |  |  |  |  |  |  |  |  |
| **Comorbidities** |  | Number of Comorbidities | 0.045 | 0.173 | 0.793 | 1.047 | 0.742 | 1.476 |
|  |  |  |  |  |  |  |  |  |
| **Time since treatment** |  | Less than 1 year | 0.504 | 0.788 | 0.524 | 1.656 | 0.346 | 7.935 |
| **(ref: Still having treatment)** |  | More than 1 year | 0.369 | 0.545 | 0.500 | 1.447 | 0.489 | 4.280 |
|  |  | Active surveillance | -0.500 | 1.005 | 0.621 | 0.606 | 0.081 | 4.536 |
|  |  |  |  |  |  |  |  |  |
| **Treatment** |  | Number of Treatments | -0.096 | 0.324 | 0.767 | 0.908 | 0.475 | 1.736 |
|  |  |  |  |  |  |  |  |  |
| **Employment (ref: Working)** |  | Not working | -1.639 | 0.609 | 0.009 | 0.194 | 0.058 | 0.652 |
|  |  |  |  |  |  |  |  |  |
| **Education (ref: No Qualifications)** |  | GCSE/Vocational | 0.718 | 0.666 | 0.286 | 2.051 | 0.540 | 7.789 |
|  |  | A level | -0.308 | 0.894 | 0.732 | 0.735 | 0.123 | 4.399 |
|  |  | University education | 0.926 | 0.610 | 0.133 | 2.525 | 0.748 | 8.523 |
|  |  |  |  |  |  |  |  |  |
| **Ethnicity (ref: White)** |  | Ethnic Minority | 0.452 | 0.815 | 0.580 | 1.572 | 0.310 | 7.959 |

Note: ref = Reference category. *Significant at *p<.05;* **Significant at *p<.01*

***Table S3: Logistic Regression Coefficients and Odds Ratios for Meeting MVPA Guidelines Stratified by Cancer Type (Colorectal Cancer)***

|  |  |  | **Coefficient** | **Std Error** | ***p*** | **OR** | **LC95%** | **UC95%** |
| --- | --- | --- | --- | --- | --- | --- | --- | --- |
| **BMI (ref: Underweight/healthy weight)** |  | Overweight | 0.379 | 0.475 | 0.427 | 1.461 | 0.568 | 3.757 |
|  |  | Obese | -0.215 | 0.562 | 0.703 | 0.807 | 0.264 | 2.461 |
|  |  |  |  |  |  |  |  |  |
| **Comorbidities** |  | Number of Comorbidities | -0.152 | 0.180 | 0.403 | 0.859 | 0.600 | 1.231 |
|  |  |  |  |  |  |  |  |  |
| **Time since treatment** |  | Less than 1 year | 0.243 | 0.588 | 0.680 | 1.275 | 0.396 | 4.110 |
| **(ref: Still having treatment)** |  | More than 1 year | 0.742 | 0.540 | 0.173 | 2.101 | 0.716 | 6.160 |
|  |  | Active surveillance | -0.938 | 1.255 | 0.457 | 0.392 | 0.032 | 4.726 |
|  |  |  |  |  |  |  |  |  |
| **Treatment** |  | Number of Treatments | 0.003 | 0.267 | 0.992 | 1.003 | 0.589 | 1.707 |
|  |  |  |  |  |  |  |  |  |
| **Employment (ref: Working)** |  | Not working | 0.129 | 0.466 | 0.783 | 1.137 | 0.451 | 2.867 |
|  |  |  |  |  |  |  |  |  |
| **Education (ref: No Qualifications)** |  | GCSE/Vocational | 0.649 | 0.601 | 0.284 | 1.913 | 0.576 | 6.350 |
|  |  | A level | -0.328 | 0.796 | 0.682 | 0.720 | 0.148 | 3.516 |
|  |  | University education | 0.599 | 0.607 | 0.328 | 1.820 | 0.540 | 6.128 |
|  |  |  |  |  |  |  |  |  |
| **Ethnicity (ref: White)** |  | Ethnic Minority | -0.838 | 0.768 | 0.278 | 0.433 | 0.094 | 1.990 |

Note: ref = Reference category. *Significant at *p<.05;* **Significant at *p<.01*
